# The impact of the stress hyperglycemia ratio on the risk of contrast-associated acute kidney injury in patients undergoing coronary angiography: a large real-world cohort study

**DOI:** 10.1186/s13098-024-01345-5

**Published:** 2024-05-21

**Authors:** Yuqi Li, Liting Zhang, Weiqi Liu, Jingru Deng, Jin Liu, Yang Zhou, Li Feng, Jiyan Chen

**Affiliations:** 1 Department of Cardiology, Zhongshan City People’s Hospital, Zhongshan, 528400 China; 2 Department of Cardiology, Guangdong Cardiovascular Institute, Guangdong Provincial People’s Hospital, Guangdong Academy of Medical Sciences, Guangzhou, 510080 China; 3 Guangdong Provincial Key Laboratory of Coronary Heart Disease Prevention, Guangdong Cardiovascular Institute, Guangdong Provincial People’s Hospital, Guangdong Academy of Medical Sciences, Guangzhou, 510080 China

**Keywords:** Contrast-associated acute kidney injury, Stress hyperglycemia ratio, Coronary angiography

## Abstract

**Background:**

Contrast-associated acute kidney injury (CA-AKI) is an important complication in the perioperative period of coronary angiography (CAG). Dysglycemia is closely associated with the occurrence of CA-AKI. However, the association between stress hyperglycemia and CA-AKI in patients undergoing CAG remains unclear. The study aims to investigate the association of the stress hyperglycemia ratio (SHR) and CA-AKI under CAG in a large real-world cohort.

**Methods:**

This was a retrospective observational study, and patients undergoing CAG were enrolled. SHR is calculated by dividing the random blood glucose with the estimated average glucose derived from the glycosylated hemoglobin (HbA1c), and subjects were divided into five groups according to SHR. The outcome was CA-AKI defined as an increase in serum creatinine of ≥ 0.3 mg/dL (26.5 μmol/L) or 1.5-fold higher than normal levels in 48 h. The association was assessed with logistic regression and restricted cubic spline analysis.

**Results:**

In 19,965 participants (men: 73.3%, mean age: 63.1 ± 10.8 years) undergoing CAG, a total of 1,621 CA-AKI cases occurred. There were reverse J-shaped associations between the SHR and CA-AKI after adjustment for other confounding factors. Moreover, SHR improved the predictive effectiveness of the traditional Mehran score (AUC 0.65 vs 0.63, *P* < 0.001), a predictive model of CA-AKI in patients undergoing percutaneous coronary intervention.

**Conclusions:**

There were reverse J-shaped associations of SHR with CA-AKI risk among patients undergoing CAG, and the assessment of SHR before CAG may assist clinicians in identifying patients at higher risk of CA-AKI.

## Introduction

As the burden of cardiovascular disease increases, the number of coronary angiographies (CAGs) continues to rise [1]. Contrast-associated acute kidney injury (CA-AKI) is a commonly occurring complication of CAG [2, 3], the incidence of which is reported to range from 3 to 50% [4–6] and is associated with adverse prognosis, including major adverse cardiovascular events, mortality, end-stage renal disease (ESRD) and prolonged hospitalization [7–10].

Previous studies have shown that hyperglycemia at admission or before the procedure is independently associated with a greater risk of CA-AKI in acute myocardial infarction (AMI) patients [3, 11]. However, hyperglycemia at admission results not only from acute stress conditions but also from poor chronic glycemic control. Stress hyperglycemia refers to transient hyperglycemia during illness under physical and/or psychological stress [12], presenting a critical or stressed status and reflecting the actual glycometabolic status. Some studies have proposed the stress hyperglycemia ratio (SHR), a novel index using random blood glucose (RBG) divided by the estimated average glucose, to quantify stress hyperglycemia [13]. However, the association between actual glucose status and CA-AKI in the overall CAG population, which includes non-AMI patients, remains unclear, and there is currently a lack of tools to assess CA-AKI risk in these patients.

Correspondingly, we sought to assess the relationship between SHR and subsequent CA-AKI risk in patients referred for CAG to assist clinicians in identifying patients at high risk for CA-AKI.

## Methods

### Study population

**Fig. 1:**
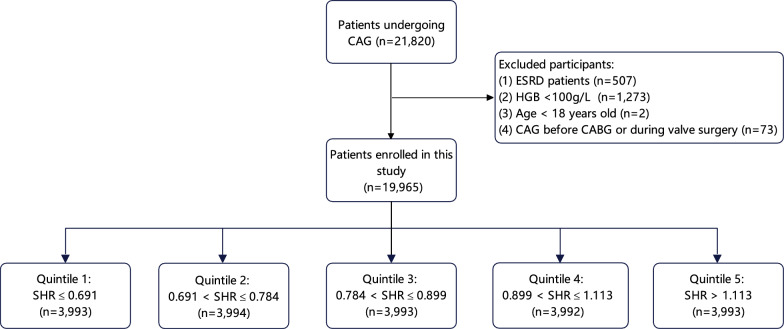
Patient flowchart. CAG: coronary angiography; ESRD: end-stage renal disease; HGB: hemoglobin; CABG: coronary artery bypass grafting; SHR: stress hyperglycemia ratio

This study aimed to investigate the association of SHR and CA-AKI under CAG in a large real-world cohort. The Cardiorenal ImprovemeNt II (CIN-II, NCT05050877) study is a large-scale retrospective cohort study that was conducted in five large tertiary hospitals in China from January 2007 to December 2020. Patients who underwent CAG were consecutively screened (n = 21,820). Patients meeting the following criteria were included: (1) patients undergoing CAG, (2) available RBG and HbA1c data, and (3) available creatines before and within 48 h after angiography. Patients meeting the following criteria were excluded: (1) admission hemoglobin < 100 g/L, (2) ESRD defined as maintenance dialysis or an estimated glomerular filtration rate (eGFR) < 15 ml/min/1.73m^2^, (3) age < 18 years and (4) patients undergoing CAG before coronary artery bypass graft or during valve surgery. Finally, 19,965 patients were successfully enrolled. The population enrollment process is illustrated in Fig. 1.

The study was approved by the Ethics Committee of Guangdong Provincial People’s Hospital (No. GDREC2019-555H-2) and all participant hospitals and was conducted according to the Declaration of Helsinki. Informed consent was waived by our committee because of the retrospective nature of our study.

### Data collection

Data were derived from the Electronic Clinical Management System (ECMS), which included demographic information, medical history, laboratory examination, procedure, medication and discharge status. RBG was measured using a standardized biochemical assay. HbA1c was routinely tested with high-performance liquid chromatography. The estimated average chronic glycemic level was calculated with the formula [(28.7 × HbA1c %) − 46.7] [13], and SHR was defined as glucose on admission divided by the estimated average chronic glycemic value. The eGFR was calculated using the Chronic Kidney Disease Epidemiology Collaboration (CKD-EPI) equation [14]. The left ventricular ejection fraction (LVEF) was measured using the biplane Simpson method with echocardiography.

### Outcomes and definition

The primary endpoint of this study was CA-AKI, defined as an increase in serum creatinine (Scr) of ≥ 0.3 mg/dL (26.5 μmol/L) or 1.5-fold higher than baseline 48 h after CAG according to the Acute Kidney Injury Network (AKIN) classification [15]. CAD and acute myocardial infarction (AMI) were confirmed by coronary angiography and discriminated according to the 10th Revision Codes of the International Classification of Diseases. DM was defined as either known diabetes (defined as ongoing medical treatment for diabetes [insulin or antidiabetics]) or newly diagnosed diabetes (defined as hemoglobin A1c level ≥ 6.5%). Chronic kidney disease (CKD) was defined as an eGFR < 60 ml/min/1.73m^2^. Anemia was defined as a baseline hematocrit value of < 39% for men and < 36% for women [16]. Congestive heart failure (CHF) was defined as New York Heart Association functional class > 2 or Killip class > 1 [17]. The Mehran score is a clinical predictor of the risk of AKI in patients undergoing percutaneous coronary intervention (PCI) [16], which brings hypotension, intra-aortic balloon pump (IABP) use, CHF, eGFR, age, anemia, DM and contrast medium volume (CMV) into the model. The controlling nutritional status (CONUT) score is a system for nutritional screening, which is calculated by three laboratory values: plasma albumin, plasma cholesterol and total lymphocyte count [18]. The score has been proven to be an independent risk factor for CA-AKI in CHF patients following CAG in previous research by our team [19].

### Statistical analysis

Patients were stratified into 5 groups based on the SHR level. Continuous variables were expressed as the mean ± standard deviation (SD) or median with interquartile range. Categorical variables were described as a number (n) with percentage (%). Differences were assessed using analysis of variance or the Kruskal–Wallis H test for continuous variables and Pearson’s χ^2^ test for categorical variables. Logistic regression analysis was used to assess the association between SHR and CA-AKI. Model 1 was unadjusted. Model 2 was adjusted for age (as a continuous variable) and gender. The clinically relevant factors and unevenly distributed variables among groups were enrolled in model 3, including age, sex, smoking status, PCI, Mehran score, CONUT score, LDLC, valvular heart disease, and critical illness. Unadjusted and adjusted odds ratios (ORs) with 95% confidence intervals (CIs) were calculated. In addition, restricted cubic spline (RCS) analyses with three knots were performed to explore the characteristics of the correlation between SHR and CA-AKI. In the RCS model, confounding factors as mentioned above were also adjusted. We conducted subgroup analyses of patients stratified by age, sex, history of CHF and DM, eGFR and diagnosis of CAD and AMI, and interaction analysis was further performed to examine whether the effects of SHR differed across different subgroups. The improvement of the Mehran score for AKI prediction by adding SHR to the model was identified using a receiver operating characteristic (ROC) curve analysis, and the nonparametric approach of DeLong et al. was used to analyze differences between ROC curves [20]. All tests were 2-tailed, and *P* < 0.05 was considered significant. The statistical analyses were performed using R version 4.3.0.

## Results

### Baseline characteristics

Table 1 summarizes the characteristics of the study population according to the quintiles of the SHRs. A total of 19,965 patients undergoing CAG were enrolled in our study. The mean ± SD age was 63.1 ± 10.8 years, 5,323 (26.7%) patients were women, 15,168 (76.0%) patients were identified as having CAD, 10,972 (55.0%) patients suffered from hypertension, 3,499 (17.5%) subjects were diagnosed with CHF, 7,541 (37.8%) were diagnosed with diabetes and 5,276 (26.4%) were diagnosed with CKD. A total of 4,348 (21.8%) patients were critically ill. We found that the youngest population was in quintile 3, where patients tended to have better cardiac function and a lower incidence of CAD, CKD and stroke. Moreover, patients in quintile 2 were more likely to have CA-AKI (10.5%), despite better renal function (eGFR 78.4 ± 33.6 ml/min/1.73m^2^) and fewer critical cases (18.4%). The contrast used in angiography includes iso-osmolality (iodixanol, N = 1523) and low-osmolality (ioversol, iohexol, iopamidol and iopromide, N = 15,432) contrast medium.

**Table 1:** Patient baseline characteristics according to quintiles of stress hyperglycemia ratio

	Overall *N* = 19,965	Quintile 1 SHR ≤ 0.691 *N* = 3,993	Quintile 2 0.691 < SHR ≤ 0.784 *N* = 3,994	Quintile 3 0.784 < SHR ≤ 0.899 *N* = 3,993	Quintile 4 0.899 < SHR ≤ 1.113 *N* = 3,992	Quintile 5 SHR > 1.113 *N* = 3,993	*P* Value
*a. General and clinical data*							
Age, years	63.1 (10.8)	63.0 (10.9)	63.0 (10.5)	62.6 (10.8)	63.3 (11.1)	63.7 (10.7)	< 0.001
Female	5323 (26.7)	1089 (27.3)	1022 (25.6)	1083 (27.1)	1068 (26.8)	1061 (26.6)	0.465
Smoke	6607 (37.7)	1295 (36.4)	1325 (37.9)	1293 (36.2)	1353 (38.9)	1341 (39.1)	0.025
CA-AKI	1621 (8.1)	320 (8.0)	420 (10.5)	311 (7.8)	254 (6.4)	316 (7.9)	< 0.001
CAD	15,168 (76.0)	2944 (73.7)	2958 (74.1)	2888 (72.3)	3108 (77.9)	3270 (81.9)	< 0.001
Diabetes	7541 (37.8)	2030 (50.8)	1152 (28.9)	1190 (29.8)	1390 (34.8)	1779 (44.6)	< 0.001
Critical illness	4348 (21.8)	798 (20.0)	736 (18.4)	739 (18.5)	997 (25.0)	1078 (27.0)	< 0.001
Hypertension	10,972 (55.0)	2177 (54.5)	2210 (55.3)	2164 (54.2)	2185 (54.7)	2236 (56.0)	0.509
Anemia	6710 (33.6)	1248 (31.3)	1292 (32.3)	1145 (28.7)	1448 (36.3)	1577 (39.5)	< 0.001
Atrial fibrillation	1662 (8.3)	296 (7.4)	408 (10.2)	342 (8.6)	307 (7.7)	309 (7.7)	< 0.001
Stroke	1262 (6.3)	230 (5.8)	264 (6.6)	229 (5.7)	241 (6.0)	298 (7.5)	0.006
Hyperlipemia	13,126 (65.7)	2598 (65.1)	2677 (67.0)	2572 (64.4)	2622 (65.7)	2657 (66.5)	0.091
CHF	3499 (17.5)	642 (16.1)	629 (15.7)	506 (12.7)	790 (19.8)	932 (23.3)	< 0.001
CKD	5276 (26.4)	955 (23.9)	1047 (26.2)	933 (23.4)	1081 (27.1)	1260 (31.6)	< 0.001
Valvular heart disease	2606 (13.1)	507 (12.7)	613 (15.3)	562 (14.1)	485 (12.1)	439 (11.0)	< 0.001
IABP use	700 (3.5)	98 (2.5)	116 (2.9)	85 (2.1)	160 (4.0)	241 (6.0)	< 0.001
Mehran score	8.1 (4.3)	7.5 (4.1)	8.7 (4.2)	7.2 (3.9)	8.2 (4.4)	9.0 (4.7)	< 0.001
CONUT score	2.3 (2.0)	2.1 (1.9)	2.1 (1.8)	2.0 (1.8)	2.4 (2.0)	2.7 (2.1)	< 0.001
*b. Laboratory analyses*							
eGFR, mL/min/1.73m^2^	76.1 (27.3)	77.1 (24.3)	78.4 (33.6)	78.3 (27.9)	74.8 (24.6)	72.1 (24.1)	< 0.001
Hemoglobin, g/L	133.9 (15.2)	134.7 (15.0)	134.1 (15.3)	135.2 (14.8)	133.4 (15.1)	132.1 (15.4)	< 0.001
RBG, mmol/L	7.0 (3.0)	5.0 (1.1)	5.5 (1.2)	6.2 (1.5)	7.5 (2.2)	10.8 (3.7)	< 0.001
LDLC, mmol/L	2.8 (0.9)	2.8 (1.0)	2.8 (0.9)	2.8 (0.9)	2.8 (0.9)	2.8 (1.0)	0.701
HDLC, mmol/L	1.0 (0.3)	1.0 (0.3)	1.0 (0.3)	1.0 (0.3)	1.0 (0.3)	1.0 (0.3)	0.002
hs-TnT, ng/L	20.2 [9.4, 133.4]	17.1 [8.6, 76.9]	16.3 [9.0, 43.3]	14.5 [8.0, 45.9]	27.6 [10.5, 440.8]	45.6 [13.1, 641.2]	< 0.001
NT-ProBNP, pg/mL	383.0 [91.4, 1538.0]	327.3 [74.8, 1359.0]	323.0 [90.1, 1305.2]	252.3 [67.1, 1103.0]	493.8 [110.8, 1862.0]	710.4 [141.1, 2569.5]	< 0.001
LVEF, %	57.5 (13.1)	58.1 (12.6)	57.8 (13.2)	59.1 (12.5)	56.8 (13.3)	55.4 (13.5)	< 0.001
*c. Therapy*							
CMV, mL	127.0 (90.0)	126.1 (90.0)	127.4 (92.1)	124.1 (88.1)	129.9 (90.8)	127.3 (88.9)	0.110
ACEI/ARB	12,844 (66.0)	2558 (65.7)	2526 (64.6)	2489 (63.6)	2666 (68.4)	2605 (67.5)	< 0.001
Beta blocker	14,646 (75.2)	2890 (74.3)	2861 (73.1)	2891 (73.9)	2976 (76.3)	3028 (78.5)	< 0.001
Statins	16,008 (82.2)	3184 (81.8)	3164 (80.9)	3146 (80.4)	3246 (83.3)	3268 (84.7)	< 0.001
DAPT	12,697 (65.2)	2476 (63.6)	2471 (63.2)	2415 (61.7)	2655 (68.1)	2680 (69.5)	< 0.001
Aspirin	15,042 (77.2)	2959 (76.0)	2934 (75.0)	2920 (74.6)	3089 (79.2)	3140 (81.4)	< 0.001
OAD	3663 (18.8)	582 (15.0)	910 (23.3)	503 (12.9)	715 (18.3)	953 (24.7)	< 0.001
Insulin	417 (2.1)	54 (1.4)	82 (2.1)	45 (1.2)	89 (2.3)	147 (3.8)	< 0.001

Values are mean ± SD, n (%), or median (interquartile range)

SHR: stress hyperglycemia ratio; CA-AKI: contrast-associated acute kidney injury; CAD: coronary artery disease; CHF: congestive heart failure; CKD: chronic kidney disease; IABP: intra-aortic balloon pump; CONUT score: controlling nutritional status score; eGFR: estimated glomerular filtration rate; RBG: random blood glucose; LDLC: low density lipoprotein cholesterol; HDLC: high density lipoprotein cholesterol; LVEF: left ventricular ejection fraction; CMV: contrast medium volume; ACEI: angiotensin-converting enzyme inhibitors; ARB: angiotensin receptor blockers; DAPT: dual antiplatelet therapy; OAD: oral antidiabetic drugs

### Association of SHR and CA-AKI occurrence

The crude and adjusted associations between SHR and CA-AKI is presented in Table 2. When SHR was considered a continuous variable, a higher level of SHR was associated with a lower rate of CA-AKI (OR, 0.64, 95% CI, 0.51–0.80, *P* < 0.001) after adjusting for all covariates (Table 2, Continuous). The trend appeared to be nonlinear when we categorized individuals by SHR quintiles: the highest risk of CA-AKI was observed in the first SHR subgroup after adjusting for all covariates (OR, 1.85, 95% CI 1.50–2.29, *P* < 0.001). In model 3, the ORs for CA-AKI comparing quintile 2 and quintile 3 with quintile 4 were 1.58 (95% CI 1.27–1.97, *P* < 0.001) and 1.49 (95% CI 1.20–1.85, *P* < 0.001), respectively, while there was no significant difference in OR between quintile 5 and quintile 4 (OR, 1.21, 95% CI 0.97–1.51, *P* = 0.086) (Table 2, Categorical 1).

**Table 2:** Univariate and multivariate logistic regression analyses for the association between SHR level and CA-AKI

	Model 1		Model 2		Model 3	
	OR (95% CI)	*P* for trend	OR (95% CI)	*P* for trend	OR (95% CI)	*P* for trend
Continuous	0.80 (0.67–0.95)	0.012	0.78 (0.65–0.93)	0.006	0.64 (0.51–0.80)	< 0.001
*Categorical 1*						
SHR ≤ 0.691	1.73 (1.47–2.04)	< 0.001	1.75 (1.49–2.06)	< 0.001	1.85 (1.50–2.29)	< 0.001
0.691—0.784	1.24 (1.05–1.48)	0.013	1.26 (1.06–1.50)	0.008	1.58 (1.27–1.97)	< 0.001
0.784—0.899	1.28 (1.08–1.52)	0.004	1.29 (1.09–1.53)	0.003	1.49 (1.20–1.85)	< 0.001
0.899—1.113	Reference		Reference		Reference	
SHR > 1.113	1.27 (1.07–1.50)	0.007	1.26 (1.06–1.49)	0.009	1.21 (0.97–1.51)	0.086
*Categorical 2*						
SHR ≤ 1.153	1.03 (0.90–1.19)	0.939	1.05 (0.92–1.20)	0.505	1.21 (1.01–1.44)	0.038
SHR > 1.153	Reference		Reference		Reference	

(Continuous) Odds ratio of CA-AKI with SHR as a continuous variable. (Categorical 1) Odds ratio of CA-AKI with SHR as a categorical variable. SHR was modeled as a categorical variable with cut points of 0.691, 0.784, 0.899 and 1.113. (Categorical 2) Odds ratio of CA-AKI with SHR ≤ 1.153 compared with patients with SHR > 1.153. Model 1: unadjusted. Model 2: adjusted for age and gender. Model 3: adjusted for age, gender, smoke, PCI, Mehran score, CONUT score, LDLC, valvular heart disease, and critical illness

**Fig. 2:**
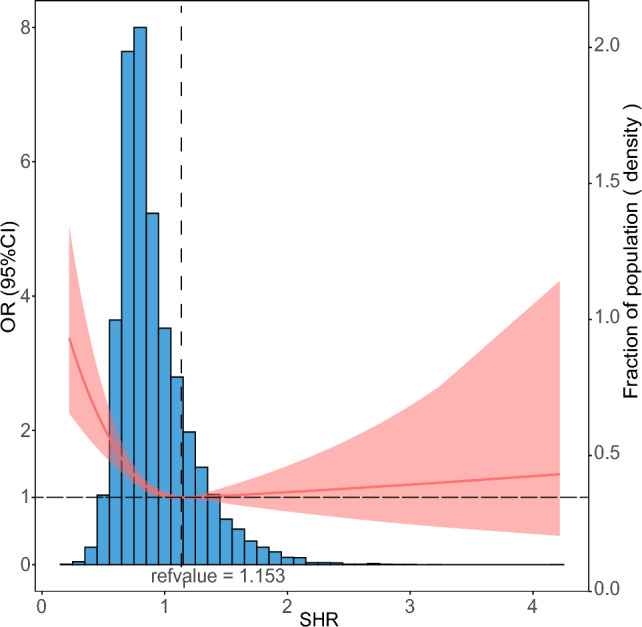
Association of SHR and CA-AKI. The analysis was adjusted for confounding factors, including age, sex, smoking status, PCI, Mehran score, CONUT score, LDLC, valvular heart disease, and critical illness. ORs are indicated by red solid lines, and 95% CIs are indicated by red shadow areas. Density distribution plots are presented by blue shadow areas. OR: odds ratio; PCI: percutaneous coronary intervention. CONUT score: controlling nutritional status score; LDLC: low-density lipoprotein cholesterol

Figure 2 shows the restricted cubic spline of the CA-AKI risk across levels of SHR. The curve revealed that there were reverse J-shaped associations between SHR and CA-AKI after adjusting for other confounding factors. The value of the SHR corresponding to the lowest risk of CA-AKI in the multivariate-adjusted RCS analyses was 1.153. Logistic regression was then performed to calculate the OR of SHR by categorizing subjects by the inflection point, and the risk of CA-AKI increased by 21% when SHR < 1.153 (OR, 1.21, 95% CI 1.01–1.44, *P* = 0.038) (Table 2, Categorical 2).

### Subgroup analysis of associations between SHR and CA-AKI

**Fig. 3:**
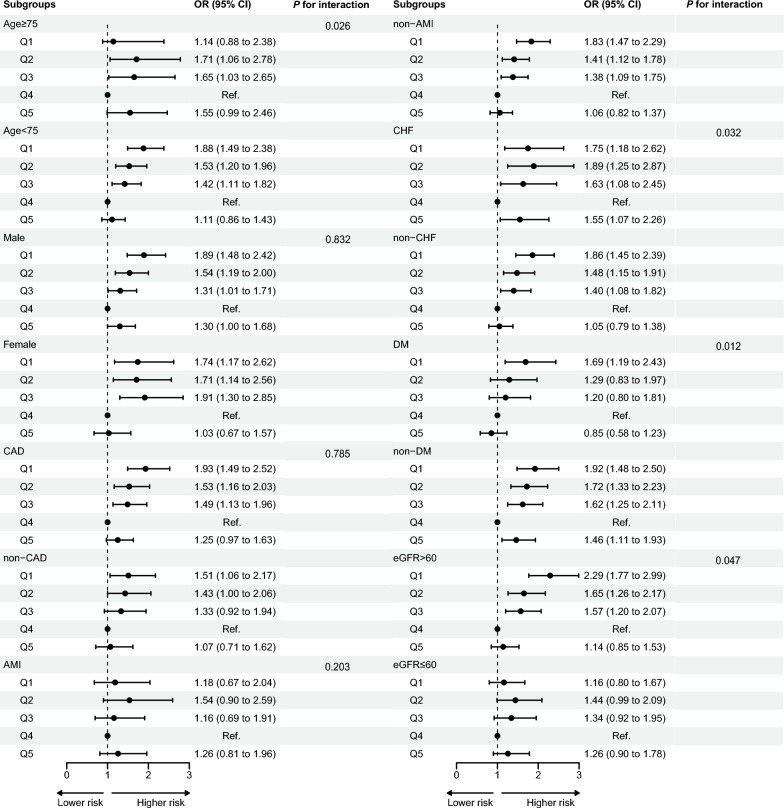
Subgroup analyses for the association between SHR levels and CA-AKI. Subgroup analyses were conducted by stratifying different variables, including age, sex, CAD, AMI, CHF, DM, and eGFR. CAD: coronary artery disease; AMI: acute myocardial infarction; CHF: congestive heart failure; DM: diabetes mellitus; eGFR: estimated glomerular filtration rate

Figure 3 elaborates the subgroup analysis of associations between SHR and CA-AKI risk. Patients were stratified by age, sex, history of CHF and DM, eGFR and diagnosis of CAD and AMI. Reverse J-shaped associations between SHR and CA-AKI were observed in younger patients, non-CHF patients, non-DM patients, non-AMI patients and patients with normal renal function but not in elderly patients, CHF patients, DM patients, AMI patients or patients with renal insufficiency. Nevertheless, age, CHF, DM and renal function had interactive effects on the association between SHR and CA-AKI risk (all *P* for interaction < 0.05) (Fig. 3).

### Improvement of the predictive power of the Mehran score by adding SHR to

**Fig. 4:**
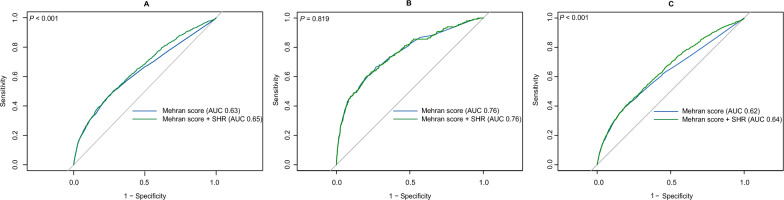
Predictive value of Mehran score ± SHR for CA-AKI in patients undergoing CAG. Receiver-operating characteristic curves showing the predictive value of Mehran score and the combined risk model in CAG patients. **A** General population. **B** AMI patients. **C** Non-AMI patients. CA-AKI: contrast-associated acute kidney injury. SHR: stress hyperglycemia ratio. AUC: area under the curve

Figure 4A compares the ROC curves for CA-AKI predictions using the Mehran score and the score combined with the SHR. The analysis indicated that the discriminatory power of the Mehran score for CA-AKI prediction in the study was improved by adding SHR (AUC 0.65 vs 0.63, *P* < 0.001). Similar effect was observed in non-AMI population (AUC 0.64 vs 0.62, *P* < 0.001), despite no significant improvement in predictive power for CA-AKI in AMI subgroup (AUC 0.76 vs 0.76, *P* = 0.819) (Fig. 4B and C).

## Discussion

In the current study, the association between SHR and CA-AKI in patients undergoing CAG was evaluated, revealing the following findings: *1*) SHR was independently associated with the risk of CA-AKI in patients undergoing CAG, and *2*) the associations were reverse J-shaped, and the ORs for CA-AKI significantly increased when SHR was < 1.153.

Previous studies have revealed that CAG per se is a stressor for patients, even in those with selective operation [21–23]. However, the association between glycemic metabolism and renal injury has been previously described only in the AMI population. Gao et al. conducted a retrospective study of 1,215 AMI patients with DM and found that the incidence of AKI increased with increasing SHR [24], whereas in the general population of patients undergoing CAG in our study, a lower level of SHR proved to be a risk factor for CA-AKI. Distinctly different results may be due to the larger population and the fact that we adjusted for the Mehran score, which has been shown to be predictive of CA-AKI [16]. In addition, Stolker et al. enrolled 6,358 AMI patients for CAG with CA-AKI as the primary endpoint. They found that the preprocedural glucose level was a predictor of CA-AKI in AMI patients without DM but not in DM patients [11]. In our study, we used SHR instead, an index that takes the chronic glucose level into account, and showed a reverse J-shaped association between SHR and CA-AKI in non-DM patients but not in DM patients undergoing CAG. Although the background blood glucose was adjusted in the index, the association of SHR and CA-AKI was still affected by DM, mainly because DM per se is a risk factor for CA-AKI [3].

In our study, patients who presented with admission blood glucose that was lower than their background blood glucose had a higher risk of CA-AKI after CAG. The stress response, including stress hyperglycemia, occurs in those under stress and is mediated by the hypothalamic‒pituitary‒adrenal axis and the sympathoadrenal system [25]. Studies have revealed that mild-to-moderate stress hyperglycemia is protective against adverse events by upregulating cell survival factors and promoting the effectiveness of cell utilization of glucose [26, 27]. Furthermore, a low SHR represents a state of relative hypoglycemia. Although they are not totally the same, hypoglycemia has been reported to be associated with increased C-reactive protein and proinflammatory cytokines, reactive oxygen species and leukocytosis [28, 29]. Nonetheless, relative hypoglycemia indicates increased glycemic variability, which triggers inflammation leading to greater oxidative stress [30, 31]. Consequently, glycemic variability and relative hypoglycemia are likely to contribute to the pathways involved in CA-AKI progression.

The study has several clinical significance and research implications. To the best of our knowledge, this is the first cohort study to investigate the relationship between SHR and CA-AKI in patients undergoing CAG. Moreover, we evaluated the nonlinear correlation between SHR and the risk of CA-AKI and proposed a reverse J-shaped association between SHR and CA-AKI in patients undergoing CAG, which is similar to a prior study regarding SHR and adverse cardiovascular events in patients with acute coronary syndrome (ACS) [32]. In addition, prior studies have generally neglected the risk of CA-AKI in non-CAD patients undergoing CAG, although the association became attenuated in these patients in our study, presumably due to population heterogeneity.

There are several limitations to this study. First, this is an observational study. Second, the time interval between measurements of serum creatine was not prearranged and fixed, which may lead to bias in CA-AKI identification. Third, the specific type of CAD is not available due to the limitations of our database, and the association in ACS patients will be validated in further studies.

## Conclusions

SHR was independently associated with CA-AKI risk in patients undergoing CAG. The assessment of SHR prior to CAG may help clinicians identify high-risk populations and facilitate preemptive decision-making on renal protection strategies.

## Data Availability

The datasets used and/or analyzed during the current study are available from the corresponding author upon reasonable request.

## Abbreviations

CA-AKI: Contrast-associated acute kidney injury
CAG: Coronary angiography
SHR: Stress hyperglycemia ratio
AMI: Acute myocardial infarction
DM: Diabetes mellitus
eGFR: Estimated glomerular filtration rate
CHF: Congestive heart failure
